# Widespread foodborne cyclosporiasis outbreaks present major challenges.

**DOI:** 10.3201/eid0204.960413

**Published:** 1996

**Authors:** D G Colley


### Widespread Foodborne Cyclosporiasis Outbreaks Present Major Challenges

**To the Editor:** The organism now named *Cyclospora cayetanensis* was first recognized as a cause of human illness in 1977. For several years, as its taxonomy was deliberated, it was referred to as "cyanobacterium-, or coccidia-like bodies" (CLBs), or considered to be blue-green algae. In 1993, *C. cayetanensis* was reported to be a protozoan parasite, a coccidian member of the family *Eimeriidae*. To be infectious, the spherical, chlorine-resistant oocyst (8µm to 10µm) found in the feces of infected persons must sporulate in the environment, a process that, depending on conditions, takes at least several days. Upon examination by ultraviolet microscopy, *Cyclospora* oocysts autofluoresce and upon staining, they are variably acid-fast. The incubation period between infection and onset of symptoms averages approximately 1 week. *Cyclospora* infects the small intestine and usually causes watery diarrhea, with frequent stools. It can also cause loss of appetite, weight loss, stomach cramps, nausea, vomiting, fatigue, increased flatus, and low-grade fever. The duration of symptoms is often several weeks, and remitting courses spanning 1 to 2 months, with several relapses, have been reported. Cyclosporiasis is effectively treated with trimethoprim/sulfamethoxazole; however, therapy for patients who are sulfa-intolerant has not been identified.

Before 1996, only three outbreaks of *Cyclospora* infection had been reported in the United States. However, between May 1 and mid-July 1996 almost 1,000 laboratory-confirmed cases were reported to the Centers for Disease Control and Prevention (CDC). A few hospitalizations (<20) were reported, but no *Cyclospora*-related deaths were confirmed. These infections occurred in at least 15 states and Canadian provinces and the District of Columbia. Investigations of approximately 50 event-related outbreaks of diarrheal illness due to *C. cayetanensis*, as well as case-control studies of sporadic, laboratory-confirmed cases by several states, now clearly implicate consumption of fresh raspberries. Complete, high confidence level trace-backs of raspberry shipments related to more than 25 of the events have indicated that the raspberries responsible were imported from Guatemala between early May and mid-June 1996.

On June 17, 1996, CDC began hosting thrice-weekly conference calls to ensure close coordination among CDC, the U.S. Food and Drug Administration (FDA), and the many state and local health agencies investigating these widespread outbreaks and cases. The conference calls provided coordination in tracking and discussing this multifocal problem. In addition, on July 17, 1996, in Atlanta, CDC and FDA held a 1-day work-shop entitled "*cyclospora* - 1996," which was attended by more than 80 persons representing CDC, FDA, the U.S. Department of Agriculture, 16 states, one province, five cities, five universities, the Council of State and Territorial Epidemiologists, the Association of State and Territorial Public Health Laboratory Directors, the Pan American Health Organization, and the government of Canada. The participants in the investigations of *Cyclospora* shared the knowledge gained through their individual investigations of this multistate, multicountry outbreak. The goals of the workshop were to begin to formulate effective prevention strategies for *Cyclospora* infection, to discuss the strength of the evidence implicating Guatemalan raspberries, and to formulate research needs. The workshop allowed for discussions about the epidemiologic and trace-back studies conducted and speculation about where and how the raspberries became contaminated. Representatives from Texas, South Carolina, New York City, Florida, and New Jersey presented data from their respective case-control and cohort studies; CDC representatives provided an overview of the outbreaks and focused on multiple, specific trace-backs from more than 20 of the event-related outbreaks. FDA representatives discussed their roles and regulatory authority in foodborne investigations.

The workshop also addressed the array of scientific challenges concerning *C. cayetanensis*, such as clinical diagnostic techniques, protocols for detection of the organism on produce, and the basic biology of this protozoon. We do not know the infectious dose, the proportion of infected persons who have diarrhea, the proportion of diarrheal illness caused in various settings by *Cyclospora*, the existence of animal reservoirs, or the viability of the organism in different environmental conditions. It can be transmitted by water and food, and its transmission is seasonal (late spring/early summer), at least where it has been studied (primarily temperate, seasonal climates).

The poor sensitivity and specificity of current methods for diagnosis and detection of *Cyclospora* were discussed. A photomicrographic demonstration convinced the participants that currently the foremost requirement for accurate clinical diagnosis is a skilled microscopist. The status of polymerase chain reaction technologies for detection and diagnosis of *Cyclospora* was presented and discussed, including the inhibitory aspects of berry juices and the difficulty in oocyst recoveries from spiked berry samples. Participants stated the need for a bank of *Cyclospora* organisms and their DNA (molecular libraries) from different locations and outbreaks. Currently, we may not be able to take full advantage of such epidemiologically well-documented specimens; however, the technologies and tools will continue to advance, and these specimens need to be centrally banked now, to be made available when the tools are up to the task. An animal model needs to be developed, or at least explored. The uses for such a model include providing material (oocysts and other life-cycle stages) for reagent development (monoclonal antibodies) to allow studies of the organisms, the disease, immune responses, and potential environmental transmission. Such a model will facilitate the development of prevention and treatment strategies.

Ongoing investigations into how the raspberries were contaminated were discussed. The lack of sensitive and reproducible detection assays for *Cyclospora*, which does not replicate outside the human host, remains the major stumbling block in providing proof of contamination of suspected transmission vehicles. Studies were too preliminary for conclusions. Both the government of Guatemala and the producer/exporter associations were most helpful in the investigations and need to remain involved if we are to better understand what occurred in May and June of this year.

Throughout the workshop, a wider issue than the current situation with *Cyclospora* was discussed: the management of the emerging problem of widespread multistate and international foodborne outbreaks of both infectious and toxic nature. Such outbreaks are increasing and can be expected to worsen as the world moves toward a global food economy. What contaminates a particular food item on a farm, in a herd or crop, at a processing shed, or from a handler, can now cause widely distributed outbreaks, continents away, in a day. More coordination is needed on several fronts in the management of such outbreaks: 1) the development of a structured process for integration and coordination of epidemiologic studies; 2) more aggressive laboratory diagnostic training related to poorly recognized or understood emerging infections; 3) better coordination of press releases related to multistate outbreaks; 4) better understanding and clarification of the legal roles and responsibilities of federal, state, and local agencies; 5) and earlier involvement of industrial partners at all levels, including growing/processing, exporting/importing, transporting, and wholesale/retail sales. Because these types of outbreaks are likely to become international this aspect must be addressed in considering appropriate approaches.

The *Cyclospora* outbreaks of May and June 1996 underlined that without the ability to culture and grow the organisms, without a supply of the organism to develop expedient assays, without an established coordinating body to expedite agreed-upon means for dissemination of information, we, as public health officials, are called upon to provide guidance without the benefit of all the appropriate knowledge. The workshop engendered interchange and discussion on critical issues concerning what is known and unknown about *Cyclospora* and the outbreaks of cyclosporiasis during May and June 1996. The workshop also provided a forum in which it became apparent that public health officials must launch a committed effort to develop an established, coordinating system among agencies at all levels and deal with the threat of widespread, multistate/international foodborne outbreaks caused by infectious or toxic agents.

**Daniel G. Colley**
Centers for Disease Control and Prevention, Atlanta, Georgia, USA

### Identification of Cyclospora in Poultry

**To the Editor:** Human infection with the parasitic protozoa, *Cyclospora*, was first described in 1979 (1), and the organism was only recently categorized as an important gastrointestinal parasite. A single species, *Cyclospora cayetanensis*, has been described in humans (2), while most species in the genus *Cyclospora* have been described only in reptiles and rodents (3). The consumption of undercooked meat and exposure to contaminated water have been considered possible sources of human infection with *C. cayetanensis* (1,4). Coccidia were detected in drinking water in Nepal (5), and the parasite was identified in an animal species (one duck in Peru, by Zerpa et al. [6]) different from those in which it was described earlier. To determine whether a domestic animal is either a host or a reservoir for *C. cayetanensis*, we first examined feces from cats, which are hosts and reservoirs of *Toxoplasma gondii*, a coccidia causing human illness, but got negative results. Because *Cyclospora* were recently phylogenetically linked to *Eimeria mitis* and *E. tenella* (7), coccidial parasites of chickens, we investigated the presence of *Cyclospora* in poultry.

We pooled feces from approximately 600 4- to 6-week-old chickens from a poultry farm near Monterrey, Mexico, and extracted feces from the caecum of 50 6- to 8-week-old chickens from a poultry market at that location. By Percoll discontinuous-gradient centrifugation (Medina-De la Garza et al., submitted), both fecal pools were positive for coccidia, mainly *Eimeria* species and what we regarded as *C. cayetanensis* oocysts. Presence of *Cyclospora* was confirmed by 1) characteristic morphology and size (8µm to 10µm), 2) positive staining with Kinyoun's acid-fast stain, 3) positive autofluorescence under ultraviolet light, and 4) sporulation of oocysts with formation of sporocysts after a 10-day incubation. All these are diagnostic features of *C. cayetanensis* (8) and to our knowledge are not described for any known poultry coccidia.

On the basis of these findings, we suggest that poultry may serve as a possible source for human infection with *Cyclospora*. Consumption of chicken has been reported in one infected patient in the original description by Ashford (1) and in a patient reported recently by Connor and Shlim (9). Moreover, the only existing report of *C. cayetanensis* found in feces from a domestic farm animal concerned a farm duck (6). Zerpa et al. suggest that besides consumption of contaminated water, other modes of transmission involving contact with domestic animals must be considered. So far, however, a possible infection route involving poultry, whether it may be direct consumption of undercooked chicken meat, contamination of food and water sources with chicken feces, or both, remains to be determined. It should be noted that sanitary standards in poultry-breeding facilities in developing countries may not be adequate. This would account for the fact that reports implicating chickens in the transmission of *Cyclospora* (1,9) have occurred in, or in relation to, developing countries. The *Cyclospora* found in the chickens in our study have the diagnostic features of C. *cayetanensis*. Nevertheless, the existence of another, not yet described, *Cyclospora* species infecting poultry, which has similar features but is different from *C. cayetanensis*, cannot be excluded at this stage. In addition, the number of oocysts recovered was not large and because feces were pooled, we could not calculate the number of oocysts passed by each bird. The possibility that oocysts were acquired as a contaminant from food or water sources and were only passing though the gut of the chickens (making the chickens a paratonic host) cannot be ruled out.

The increased recognition of *Cyclospora* as an important cause of diarrhea in both immunocompromised and immunocompetent

